# An unusual cause of anterior wall ST-elevation myocardial infarction: a case report

**DOI:** 10.1093/ehjcr/ytae243

**Published:** 2024-05-14

**Authors:** Zouhir Dindane, Elena Golgor, Axel Linke, Norman Mangner

**Affiliations:** Department of Internal Medicine and Cardiology, Herzzentrum Dresden, Technische Universität Dresden, Fetscherstrasse 76, 01307 Dresden, Germany; Department of Diagnostic and Interventional Radiology, University Hospital Dresden, Technische Universität Dresden, Dresden, Germany; Department of Internal Medicine and Cardiology, Herzzentrum Dresden, Technische Universität Dresden, Fetscherstrasse 76, 01307 Dresden, Germany; Department of Internal Medicine and Cardiology, Herzzentrum Dresden, Technische Universität Dresden, Fetscherstrasse 76, 01307 Dresden, Germany

**Keywords:** Case report, Metastatic myocardial infiltration, Acute coronary syndrome, Cardiac imaging, Coronary intervention

## Abstract

**Background:**

Metastatic tumours, notably lung cancer, can cause conditions resembling acute myocardial infarctions (AMIs), contributing to the minor percentage of AMIs unrelated to coronary atherosclerosis. These instances necessitate specialized diagnostic and therapeutic approaches due to the distinct underlying pathology.

**Case summary:**

We report a case of a 65-year-old male with metastatic lung cancer presenting with prolonged angina pectoris. Elevated troponin and creatine kinase levels led to emergency catheterization, revealing a total occlusion of the distal left coronary artery attributed to tumour infiltration. Intervention attempts were unsuccessful, and given the palliative context, other revascularization strategies were not pursued. Two-dimensional transthoracic echocardiogram depicted tumour invasion at the heart’s apex, confirming the diagnosis. The patient passed away shortly after receiving palliative radiation therapy.

**Discussion:**

This case underscores the challenges in diagnosing and managing myocardial infiltrations from metastatic tumours mimicking AMI. It accentuates the importance of imaging studies for accurate diagnosis and the critical evaluation of intervention strategies, highlighting the need for focused palliative care in such complex scenarios.

**Learning points**

- This case illustrates the uncommon but noteworthy occurrence of myocardial infiltration due to metastatic lung cancer, resulting in coronary artery occlusion and myocardial infarction.
- The manifestation of such conditions emphasizes the criticality of including tumour invasion as a differential diagnosis, particularly for patients with advanced stages of lung cancer.
- Beyond lung cancer, it is crucial to acknowledge that various other cancers, such as lymphomas, have been well-documented for their potential to infiltrate cardiac tissue, highlighting the need for heightened awareness and consideration of cardiac infiltration across a spectrum of cancer types.

## Introduction

Primary cardiac tumours are extremely rare. In contrast, metastatic heart involvement is nearly 20 times more frequent and has been observed in autopsy series in up to one in five cancer patients.^1^ The clinical presentation varies from asymptomatic to acute presentations with chest pain, arrhythmias, and acute myocardial infarction (AMI). We report here the case of a patient who developed AMI due to total occlusion of the left anterior descending (LAD) artery due to metastatic lung cancer.

## Summary figure

**Table:** 

Time	Event
2 years ago	Diagnosis of lung cancer (cT4cN1cM1)
Over the next 2 years	Treatment involving surgery, adjuvant chemotherapy, and three rounds of radiation therapy for the bone metastasis in the left scapula
Current presentation	Transferred to the emergency department with angina pectoris
On admission	Elevated high-sensitivity troponin and CK levels on blood test
On admission	Diagnosis of ST-elevation AMI, transfer for emergency catheterization
During catheterization	Coronary angiogram shows total occlusion of the distal LAD Unsuccessful wiring attempts of the occluded vessel
During hospital stay	TTE study shows invasion of the heart apex by the metastatic tumour, with localized thickening of the apical left ventricular wall and localized wall motion asynergy
During hospital stay	Review of previous CT images confirms tumour infiltration into the left ventricular apex
During hospital stay	Decision against further revascularization strategies due to the palliative situation
Few days after hospital stay	The patient underwent radiotherapy once more but unfortunately passed away

## Case report

A 65-year-old man with known metastatic lung cancer was transferred to our emergency department due to typical angina pectoris lasting ∼8 h.

The patient first received a diagnosis of lung cancer two years ago, which was classified based on the TNM system (tumour, nodes, metastases) as cT4cN1cM1. The palliative treatment involved a combination of surgery, adjuvant chemotherapy, and three rounds of radiation therapy to address the bone metastasis in the left scapula.

**Figure 1:**
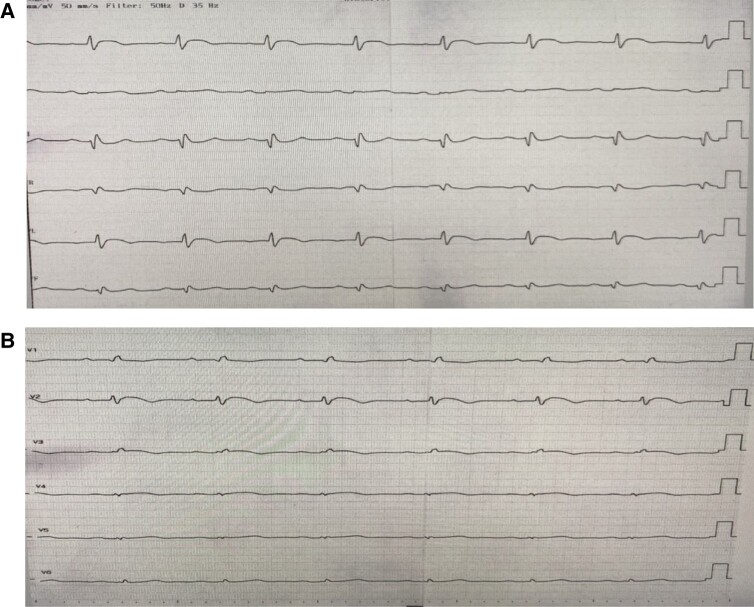
Illustrates ST-segment elevation detected in the anterolateral leads of the ECG as well as a QRS low voltage. (*A*) The limb leads; (*B*) the precordial Leads.

Until his current presentation, the patient had no history of chest pain, either at rest or during exertion, and no history of coronary artery disease. His coronary risk factors included hypertension and former smoking. On admission, the patient was haemodynamically stable without any signs of cardiac decompensation (Killip I). ECG showed ST-segment elevation in the anterolateral leads V2, V3, I, and aVL (*Figure 1*). A blood test conducted externally revealed elevated levels of high-sensitivity troponin and creatine kinase (CK), measured at 154 ng/L and 300 U/L, respectively. ST-elevation AMI was diagnosed, and the patient was transferred for emergency catheterization.

**Figure 2:**
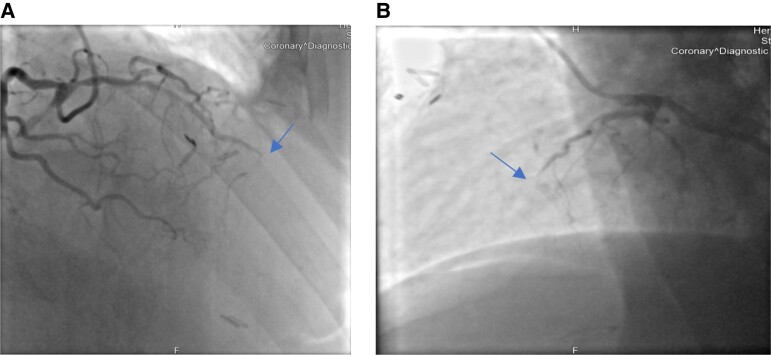
(*A*) Shows a RAO cranial view of the LCA; (*B*) shows a LAO cranial view of LCA, both views showing a distal total occlusion of the left coronary artery.

The coronary angiogram revealed a total occlusion of the distal LCA (*Figure 2*). It was conspicuous that no cardiac motion was detectable at the left ventricular apex and the mid-LCA (see Supplementary material online, *Video S1*). Several wiring attempts of the occluded vessel were unsuccessful and were not forced due the risk of perforation and bleeding under the suspicion of tumour invasion into the myocardium. Due to the palliative situation, other revascularization strategies weren’t considered.

**Figure 3:**
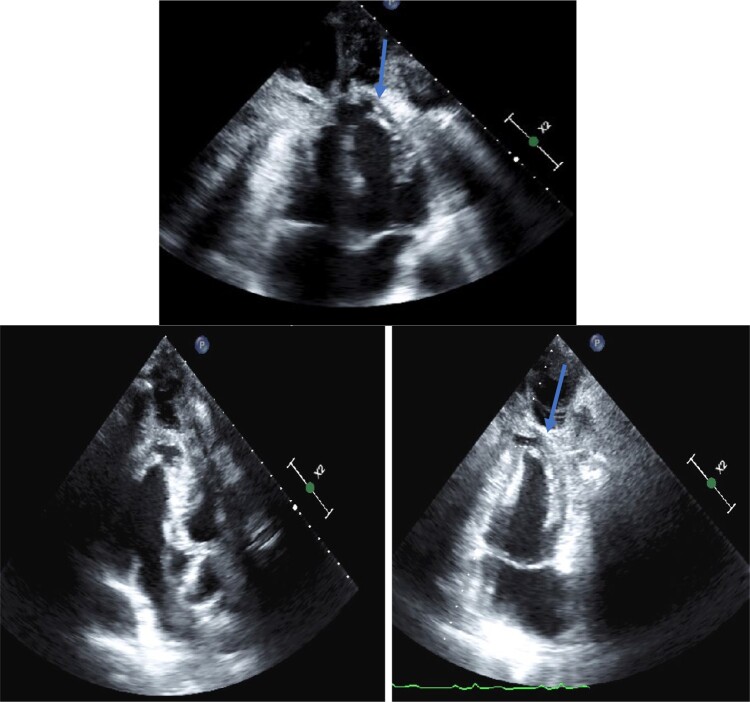
Displays a set of three views of the transthoracic echocardiogram, including a four-chamber view, a two-chamber view, and a three-chamber view. The images reveal a localized thickening of the apical left ventricular wall (arrow), which corresponds to the site of tumour attachment. The thickening is visualized as a focal area of hyperechogenity, with a blurred margin and a heterogenous texture.

**Figure 4:**
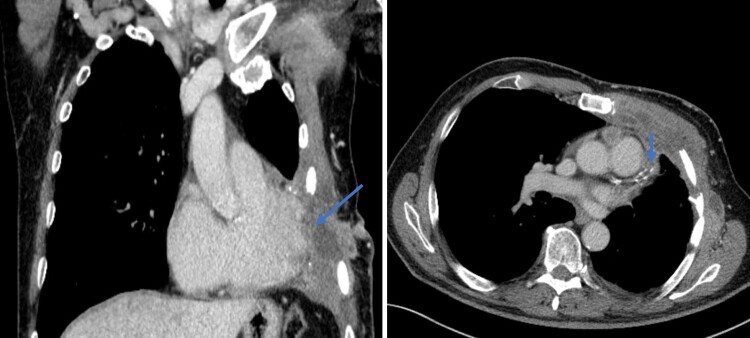
Displays a soft tissue window of a CT scan of the thorax, showing both a coronal and axial section. The images reveal a large malignant tumour infiltrating the myocardium of the left ventricle with contrast enhancement.

The performed transthoracic echocardiography (TTE) study showed an invasion of the apex of the heart by the metastatic tumour, which was manifested by localized thickening of the apical left ventricular wall along the site of tumour attachment, a localized wall motion asynergy was also observed (*Figure 3*, Supplementary material online, *Video S2*). A review of previous CT images showed evidence of tumour infiltration and contrast enhancement into the left ventricular apex (*Figure 4*). The localized thickening and wall motion asynergy observed on TTE corresponded well with the identified area of tumour involvement.

In the synopsis of the collected findings, our diagnosis hypothesis of occlusion due to tumour metastasis in the left ribs could be confirmed. The patient underwent radiotherapy once more, but unfortunately passed away a few days later.

## Discussion

About 5% of AMIs are not caused by coronary atherosclerosis and plaque rupture,^2^ and many factors are implicated, including cocaine misuse, radiation-associated fibrosis, amyloidosis, infectious illness, embolism, and neoplasia. Metastatic tumours are frequently asymptomatic, although heart failure, pericardial effusion, arrhythmia, myocardial infarction, and sudden cardiac death can happen and this relies on the location and size of the tumour.

We herein describe a case of metastatic lung cancer infiltrating the left ventricle and leading to the occlusion of the LAD artery. Given the patient’s overall condition and a life expectancy exceeding 6 months, an emergency angiogram was performed,^3^ revealing a complete occlusion of the distal LAD. Multiple attempts to wire the occluded vessel were unsuccessful. Due to the palliative situation, other revascularization strategies weren’t considered. The performed TTE study was able to delineate the location of the tumoural invasion at the apex of the heart. We decided against further imaging studies regarding the patient’s situation and the diagnosis associated with a dismal prognosis.

Myocardial involvement due to metastasis may provoke ST-elevation myocardial infarction.^4^ Such metastases might obstruct coronary arteries, either by direct invasion or compression, which could make percutaneous coronary intervention (PCI) a consideration. The appropriateness of PCI must be carefully weighed against potential bleeding risks, cancer prognosis, and the patient’s overall clinical condition^5^—including pain intensity, response to analgesia, haemodynamic stability, as well as patient and family preferences.

In assessing the tumour’s extent, imaging modalities such as two-dimensional TTE are instrumental in delineating the size and proximity of the metastasis to the coronary vessels, thus guiding the treatment strategy.

## Conclusion

We report a rare clinical scenario of a patient who had a myocardial infarction caused by the occlusion of the coronary artery by a myocardial infiltration through metastatic lung cancer. Two-dimensional echocardiography was useful for obtaining good spatial information and diagnosing the cause of AMI in this patient. A percutaneous intervention may not succeed in such cases, and palliative strategies have to be considered.

## Supplementary Material

ytae243_Supplementary_Data

## Data Availability

The data supporting the findings of this case report are fully accessible within the main text of the article and the additional online supplementary materials provided.

## Lead author biography

Dr Dindane is a resident of cardiology currently in the 6th post-graduate year of the German Board at the Heart Center Dresden. Holding a focus on heart failure and interventional cardiology. In addition to clinical practice, Dr Dindane holds a deep interest in cardiovascular research.
